# Dynamics of Response to Asynapsis and Meiotic Silencing in Spermatocytes from Robertsonian Translocation Carriers

**DOI:** 10.1371/journal.pone.0075970

**Published:** 2013-09-16

**Authors:** Anna K. Naumova, Shawn Fayer, Jacky Leung, Kingsley A. Boateng, R. Daniel Camerini-Otero, Teruko Taketo

**Affiliations:** 1 Department of Obstetrics and Gynecology, McGill University, Montreal, Quebec, Canada; 2 Department of Human Genetics, McGill University, Montreal, Quebec, Canada; 3 The Research Institute of McGill University Health Centre, Montreal, Quebec, Canada; 4 Department of Anatomy and Cell Biology, McGill University, Montreal, Quebec, Canada; 5 Genetics and Biochemistry Branch, NIDDK, NIH, Bethesda, Maryland, United States of America; 6 Department of Surgery, McGill University, Montreal, Quebec, Canada; University of Maryland School of Medicine, United States of America

## Abstract

Failure of homologous synapsis during meiotic prophase triggers transcriptional repression. Asynapsis of the X and Y chromosomes and their consequent silencing is essential for spermatogenesis. However, asynapsis of portions of autosomes in heterozygous translocation carriers may be detrimental for meiotic progression. In fact, a wide range of phenotypic outcomes from meiotic arrest to normal spermatogenesis have been described and the causes of such a variation remain elusive. To better understand the consequences of asynapsis in male carriers of Robertsonian translocations, we focused on the dynamics of recruitment of markers of asynapsis and meiotic silencing at unsynapsed autosomal trivalents in the spermatocytes of Robertsonian translocation carrier mice. Here we report that the enrichment of breast cancer 1 (BRCA1) and histone γH2AX at unsynapsed trivalents declines during the pachytene stage of meiosis and differs from that observed in the sex body. Furthermore, histone variant H3.3S31, which associates with the sex chromosomes in metaphase I/anaphase I spermatocytes, localizes to autosomes in 12% and 31% of nuclei from carriers of one and three translocations, respectively. These data suggest that the proportion of spermatocytes with markers of meiotic silencing of unsynapsed chromatin (MSUC) at trivalents depends on both, the stage of meiosis and the number of translocations. This may explain some of the variability in phenotypic outcomes associated with Robertsonian translocations. In addition our data suggest that the dynamics of response to asynapsis in Robertsonian translocations differs from the response to sex chromosomal asynapsis in the male germ line.

## Introduction

During mammalian meiosis, homologous chromosomes pair, synapse and recombine. Pairing and synapsis of homologous chromosomes is indispensable for correct chromosome segregation during meiosis and ensures that mature gametes contain a full set of chromosomes. However, in mammals, males carry sex chromosomes with homology restricted to only a small portion of their length [1,2]. The homologous regions of the sex chromosomes are termed pseudoautosomal regions and are located near the telomeres [2]. During post-zygotene stages of meiotic prophase, a special compartment, the sex body, that includes the X and Y is formed [1,3,4]. Formation of the sex body is associated with epigenetic remodeling of the sex chromatin and transcriptional repression of X- and Y -linked genes [5,6], a phenomenon termed meiotic sex chromosome inactivation (MSCI) [7-9].

Asynapsis in autosomes also triggers an epigenetic response. The most common cause of autosomal asynapsis in humans are balanced chromosomal translocations, which increase the risk of meiotic segregation errors, aneuploidy [10-12] and embryo loss. Translocation carriers are also often infertile due to meiotic arrest and failure of gametogenesis [10,11,13,14]. The detrimental effect of translocations on meiosis is attributed to meiotic silencing of genes that reside near the translocation breakpoints and are essential for meiotic progression [15]. In the male germ line, massive chromosomal asynapsis leads to reactivation of the sex chromosomes and X-linked gene expression, which also hampers male meiosis [5,16-18]. MSCI and meiotic silencing of unsynapsed chromatin (MSUC) in autosomes share a number of similarities, which led to the conclusion that MSCI is a particular case of MSUC [5,9,19]. However, it is unclear whether MSCI and autosomal MSUC are mechanistically identical.

The impact of chromosomal translocations on gametogenesis varies between sexes [20,21], individual carriers [17,22,23], and depends upon the type of translocation [10,17,24-26]. Some of this variation is explained by differences between the genes affected by different translocations [15] whereas variation between sexes is due to differences between oocyte and spermatocyte biology [20,21]. Furthermore, Robersonian translocation trivalents achieve non-homologous synapsis in a significant proportion of spermatocytes [10,25,27,28]. The efficiency of non-homologous synapsis varies with age [23], which also adds to the complexity of the phenotypic outcomes.

Importantly, heterozygous carriers of certain translocations may produce viable offspring. Moreover, with the progress in assisted reproduction techniques, subfertile translocation carriers succeed in having children [29,30]. The main concern in such cases is embryonic loss and birth defects caused by chromosomal aneuploidies resulting from abnormal chromosome segregation in meiosis [31]. In fact, about 5% of Down syndrome cases are associated with Robertsonian translocations involving chromosome 21 (reviewed in 32). Robertsonian translocations involving the acrocentric chromosomes 14 and 15 may cause uniparental disomies and therefore lead to imprinting disorders in the offspring of carriers (reviewed in 31-33). Chromosomal asynapsis and MSUC in the gametes of carriers of Robertsonian translocations may give rise to another problem: incorrect epigenetic marking of the unsynapsed chromosomal regions not only during gametogenesis, but also after conception. It has been hypothesized that the epigenetic marks of MSUC, if transmitted through the parental germ cells to embryos, may interfere with normal gene regulation, compromise embryonic development and increase the risk of congenital developmental anomalies in children [26,34]. However, the likelihood of adverse birth outcomes due to MSUC, proportions of gametes with epigenetic marks of MSUC or the stability of MSUC throughout different stages of gametogenesis remain undetermined.

Here, to determine if asynapsis in Robertsonian translocation trivalents triggers the same epigenetic response as asynapsis of sex chromosomes, we compared the localization of several MSCI markers at the autosomal trivalents and the sex chromosomes in mouse carriers of Robertsonian translocations. Our data indicate that the dynamics of recruitment of several markers of asynapsis differ between sex chromosomes and autosomal Robertsonian translocations.

## Materials and Methods

### Ethics statement

The work was approved by the McGill University Animal Care Committee (protocol #4037) and carried out in compliance with the Canadian Council on Animal Care (CCAC) guidelines.

### Mice and crosses

Mouse strains CBy.RBF-Rb(8.12) 5Bnr/J (carries a single Rb(8.12) translocation on a BALB/cBy genetic background); RBF/DnJ (carries three translocations Rb(1.3), Rb(8.12) and Rb(9.14)); and C57BL/6J were purchased from the Jackson Laboratory. The congenic strain B6.SPRET7MOLF12 was generated and maintained in our laboratory [35,36]. Heterozygous males were generated by reciprocal crosses between C57BL/6J or B6.SPRET7MOLF12 mice and homozygous carriers of translocations.

### Immunolocalization and FISH

Germ cell nuclei for immunofluorescence experiments were prepared from testes of 2 to 4 months old male mice as described in [22]. For the H3.3S31 immunostaining and FISH experiments, spermatogenic cells were squeezed out of seminiferous tubules into MEM according to Moens [37] and spun down onto histology slides after hypotonic treatment in 0.5% NaCl for 5-10 min [38].

Staging of spermatocytes was done based on the configuration of the XY bivalent and DAPI staining as earlier described [26]. Briefly, in early pachytene nuclei, the configuration of the sex chromosomal axes is fluid; synapsis may vary from minimal to maximal; DAPI staining is diffuse and the XY bivalent is often in the middle of the nucleus. In mid pachytene nuclei, the synapsed regions of the XY bivalents become shorter while the unsynapsed axes become more stiff and curved; DAPI staining is more intense around centromeres. In late pachytene nuclei, the X chromosome axis shows coils; the sex body is often on the periphery of the nucleus, and areas of intense DAPI staining around centromeric regions are more localized. Furthermore, in late pachytene spermatocytes, DAPI staining highlights the sex body as a separate structure with a more intense spot within the domain corresponding to the X (but not Y) centromere. By the end of late pachytene the X and Y may not be synapsed anymore. In early diplotene, DAPI shows further condensation around centromeres, and dissociation of some, but not all autosomal bivalents.

Immunolocalization of proteins was conducted using the following antibodies: mouse anti-γH2AX (1:1000), rabbit anti-H3K27me3 (1:200), and anti-H3K9me3 (1:500) (Millipore); mouse anti-RNA polymerase II (1:100) (Abcam, ab 24758); rabbit anti-BRCA1 (1:200) (gift of Dr. S.H. Namekawa); mouse antibodies raised against a purified synaptonemal complex (anti-SC) [39] (1:300) (gift of Dr. Peter Moens); rabbit anti-SYCP3 (1:400) (Abcam, ab 15093); rabbit anti-histone H3.3S31 (1:200) (Abcam, ab 92628); and secondary donkey anti-mouse and anti-rabbit AlexaFluor antibodies (1:500) (Invitrogen, Carlsbad, CA, USA).

Fluorescent in situ hybridization (FISH) using probes for chromosomes Y (XMPY), 8 (XMP8), and 12 (XMP12) (MetaSystems, Germany) was performed according to the manufacturer’s protocol. Most immunolocalization and all FISH data were analyzed using the Zeis Axiophot microscope. Images were captured with a digital camera (Retiga 1300, QImaging, Burnaby, BC) and processed with Northern Eclipse digital imaging software, version 6.0 (Empix Imaging, Mississauga, ON).

### Statistical analysis

Differences between translocation carriers and controls or between meiotic stages with respect to autosomal H3.3S31 or γH2AX enrichment were evaluated using Fisher’s exact test.

## Results

### Dynamics of markers of unsynapsed chromatin and unrepaired DNA, γH2AX and BRCA1, at unsynapsed autosomal regions in spermatocytes from translocation carriers

Immunolocalization of two markers of asynapsis, γH2AX and BRCA1, was assessed in spermatocytes from Robertsonian translocation carriers. Only nuclei with good chromosome spreads, unambiguous asynapsis, and unambiguous identification of translocated chromosomes were included in the data analysis. A trivalent was considered unsynapsed when a fork-like open structure was observed (Figure S1). All other configurations of the trivalent were considered synapsed (Figure S1). In carriers of three translocations, the unsynapsed chromosomes are often entangled and associated with the X chromosome. This results in unusual conformations of the sex chromosomes and the trivalents and hampers accurate staging of the pachytene spermatocytes (Figure S1). This also generates a bias in favor of nuclei with synapsed trivalents as those are easier to stage. Therefore, all analyses were conducted first in carriers of the single translocation Rb(8;12) and then immunostaining patterns were confirmed in carriers of three translocations, when possible.

**Figure 1:**
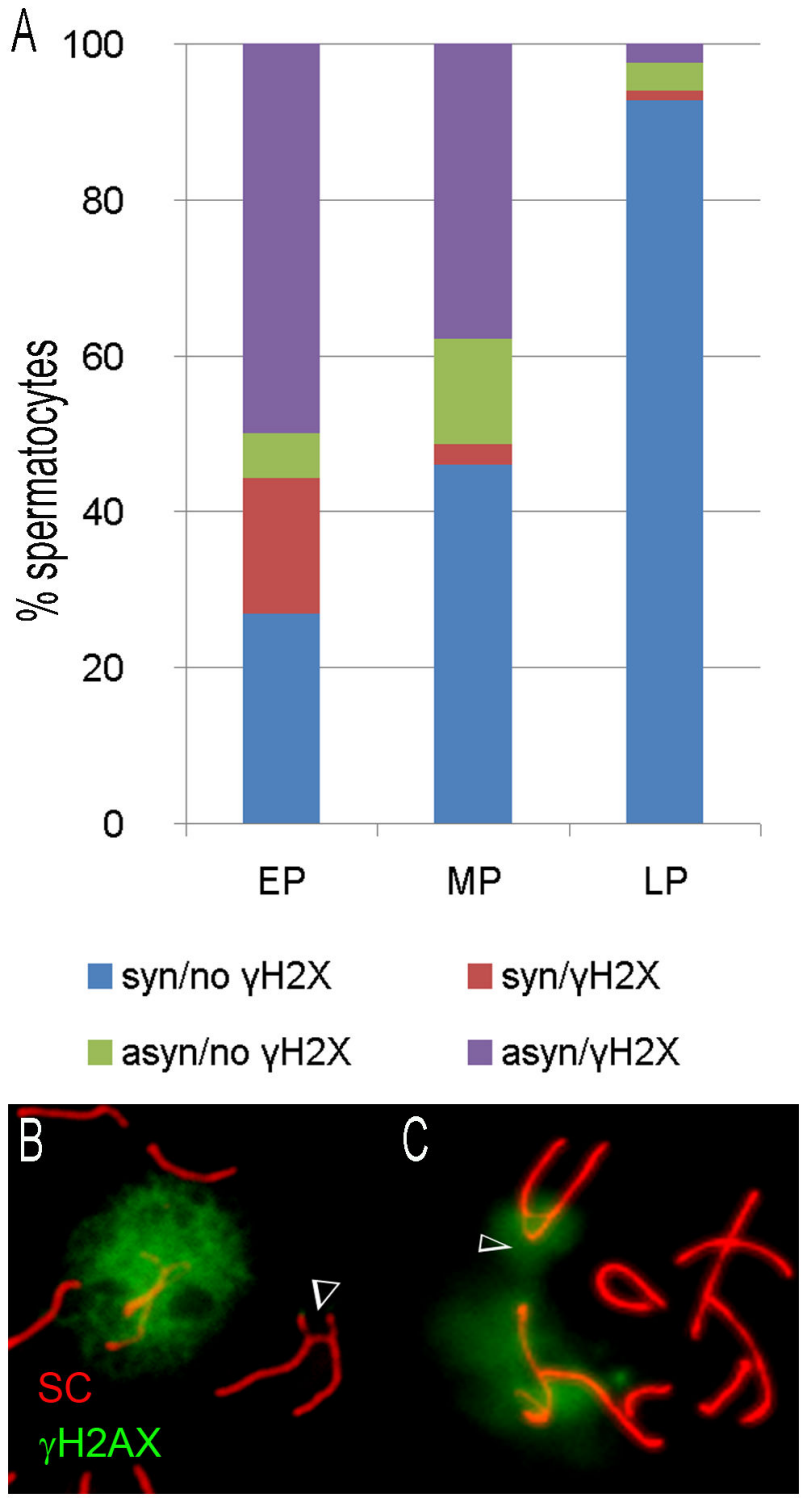
Dynamics of γH2AX localization to the chromosomal trivalents during the pachytene stage differs from the enrichment of this marker at the XY bivalent in spermatocytes of single translocation carriers. EP- early pachytene, MP – mid pachytene; LP – late pachytene spermatocytes. Arrowheads indicate trivalents. **A** - Distribution of γH2AX-positive and negative trivalents in 172 pachytene spermatocytes (from 5 mice). The y axis shows the percent spermatocytes with different γH2AX enrichment at different stages. **B** - Example of a γH2AX-negative unsynapsed trivalent in an early pachytene spermatocytes (asyn/ no γH2AX); **C** - Example of a γH2AX enrichment of a synapsing trivalent in early pachytene spermatocytes (syn/γH2AX).

In carriers of a single translocation, γH2AX enrichment was found in the pericentromeric regions of Robertsonian trivalents at all pachytene stages, however with different frequency. The proportion of spermatocytes with γH2AX enrichment at trivalents (172 nuclei counted) showed a statistically significant decline during meiotic progression from 67% at the early pachytene stage to 4% at the late pachytene stage (p= 0, Fisher’s exact test) (Figure 1A). When only unsynapsed trivalents are considered, the decline of γH2AX-positive unsynapsed trivalents from 90% at the early pachytene stage to 40% at the late pachytene stage remains statistically significant (p= 0.0287, Fisher’s exact test). A small proportion (4-13%) of spermatocytes showed unsynapsed trivalents without γH2AX immunostaining at all pachytene stages (Figure 1B), whereas 17% of early pachytene spermatocytes, and 1 to 3% at later stages, showed γH2AX-positive synapsed trivalents (Figure 1C). It is possible that in these latter cells, dephosphorylation or replacement of histone γH2AX following synapsis were not yet completed.

In summary, the population of pachytene spermatocytes is heterogeneous with respect to γH2AX localization to unsynapsed autosomal trivalents at any given stage of Pachynema. Consistent with previous reports [23,25,40], γH2AX is enriched in the pericentromeric regions of unsynapsed translocations and lost as the trivalents manage to synapse by the later stages of Pachynema.

**Figure 2:**
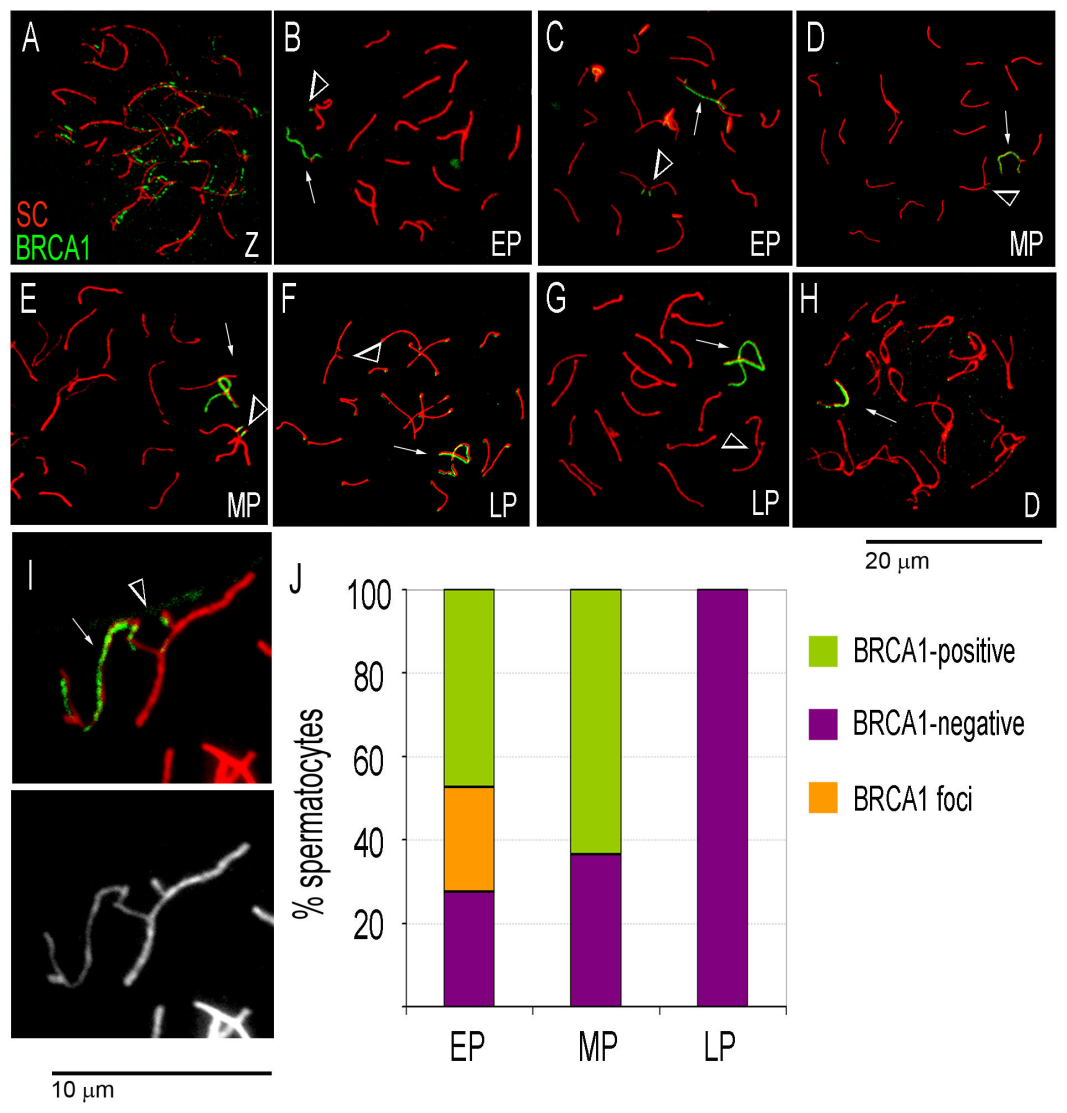
Dynamics of BRCA1 localization in meiotic prophase I spermatocytes from single translocation carriers. Z - zygotene, EP- early pachytene, MP – mid pachytene; LP – late pachytene, D-diplotene spermatocytes. Arrows point to the XY bivalents. Arrowheads indicate unsynapsed trivalents. **A** – Zygotene spermatocyte, **B** - early pachytene spermatocyte with a BRCA1-positive XY-bivalent and a single BRCA1 focus at the unsynapsed trivalent; **C** – early pachytene spermatocyte with BRCA1-positive XY-bivalent and unsynapsed trivalent; **D** – mid pachytene spermatocyte with a BRCA1-positive XY bivalent and BRCA1-negative unsynapsed trivalent; **E** - mid pachytene spermatocyte with BRCA1-positive XY-bivalent and unsynapsed trivalent; **F** – late pachytene spermatocyte with a BRCA1-positive XY bivalent and BRCA1-negative unsynapsed trivalent; **G** - late pachytene spermatocyte with a BRCA1-positive XY bivalent and BRCA1-negative synapsed trivalent; **H** – diplotene spermatocyte with a BRCA1-positive XY bivalent; **I** – BRCA1-positive XY bivalent and BRCA1 foci at the unsynapsed trivalent (enlarged 2.5 X to show detail), bottom panel shows SC-immunostaining alone. **J** – Distribution of pachytene spermatocytes with unsynapsed trivalents by stage and type of BRCA1 enrichment. The y axis shows percent spermatocytes with unsynapsed trivalents and specific BRCA1 enrichment patterns.

Next, we tested BRCA1 localization in spermatocytes (Figure 2). Three types of BRCA1 enrichment patterns were observed at trivalents: (i) BRCA1-positive unsynapsed trivalents in which both unsynapsed axes were highly enriched with BRCA1 and had the same signal intensity as the XY bivalent (Figure 2, C and E); (ii) BRCA1-negative unsynapsed trivalents with no detectable BRCA1 signal (Figure 2, D and F); and (iii) trivalents with discrete BRCA1-positive foci on the unsynapsed axes (Figure 2, B and I). All observed synapsed trivalents were BRCA1-negative (data not shown). BRCA1-positive unsynapsed trivalents were observed in early and mid, but not late, pachytene spermatocytes. BRCA1 foci were observed mostly in early pachytene spermatocytes (Figure 2). BRCA1-negative unsynapsed trivalents were observed at all stages in similar proportions. Similar to γH2AX, BRCA1 enrichment at unsynapsed trivalents declined from early to late pachytene stage: the proportion of BRCA1-positive unsynapsed trivalents was significantly higher in early (72.5%) compared to late (less than 14%) pachytene spermatocytes (p= 0.0317, Fisher’s exact test). These same BRCA1 localization patterns were also observed in carriers with three translocations (data not shown).

Our observations suggest a delayed BRCA1 recruitment to unsynapsed autosomes compared to the sex chromosomes: BRCA1 appears as discrete foci at the onset of Pachynema when the XY bivalent is already highly enriched with BRCA1; becomes abundant as the spermatocytes progress through the early pachytene and reach the mid pachytene stage; and disappears in late Pachynema, even at unsynapsed autosomal trivalents.

Overall, our data show that both BRCA1 and γH2AX are absent from unsynapsed trivalents in a small proportion of spermatocytes while retained at the sex body. Hence, if BRCA1 and γH2AX are markers of meiotic silencing, the proportion of spermatocytes with meiotic silencing declines between early and late pachytene stages in carriers of a single Robertsonian translocation Rb(8.12).

### Repressive histone marks H3K9me3 and H3K27me3 at unsynapsed trivalents

Formation of the sex body is associated with transcriptional silencing or non-reactivation of sex-chromosome linked genes [9,41,42]. Transcription in most of the spermatocyte genome is low at early pachytene and increases by the late pachytene stage [42]. The marker of transcription, RNA polymerase II (POLII), is not detected before the pachytene stage. It appears in the nucleus starting from the mid pachytene stage, but is excluded from the sex body [42]. To determine if it were also excluded from the unsynapsed trivalents, we tested its localization in carriers of one and three translocations. The POLII signal was very weak in pachytene spermatocytes and increased in late pachytene and diplotene nuclei. Furthermore, POLII seemed to be excluded from the centromeric regions of both synapsed and unsynapsed trivalents (Figure S2) making it not informative in our model.

Next, we tested the localization of two repressive chromatin marks, H3K9me3 and H3K27me3, in wild type and translocation carrier mice. H3K9me3 is associated with constitutive centromeric heterochromatin (reviewed in 43), whereas H3K27me3 is associated with facultative heterochromatin and cell-type or stage-specific transcriptional silencing [44]. Both, H3K9me3 and H3K27me3 are known to be excluded from the sex body in mid and late pachytene spermatocytes [42,45,46].

**Figure 3:**
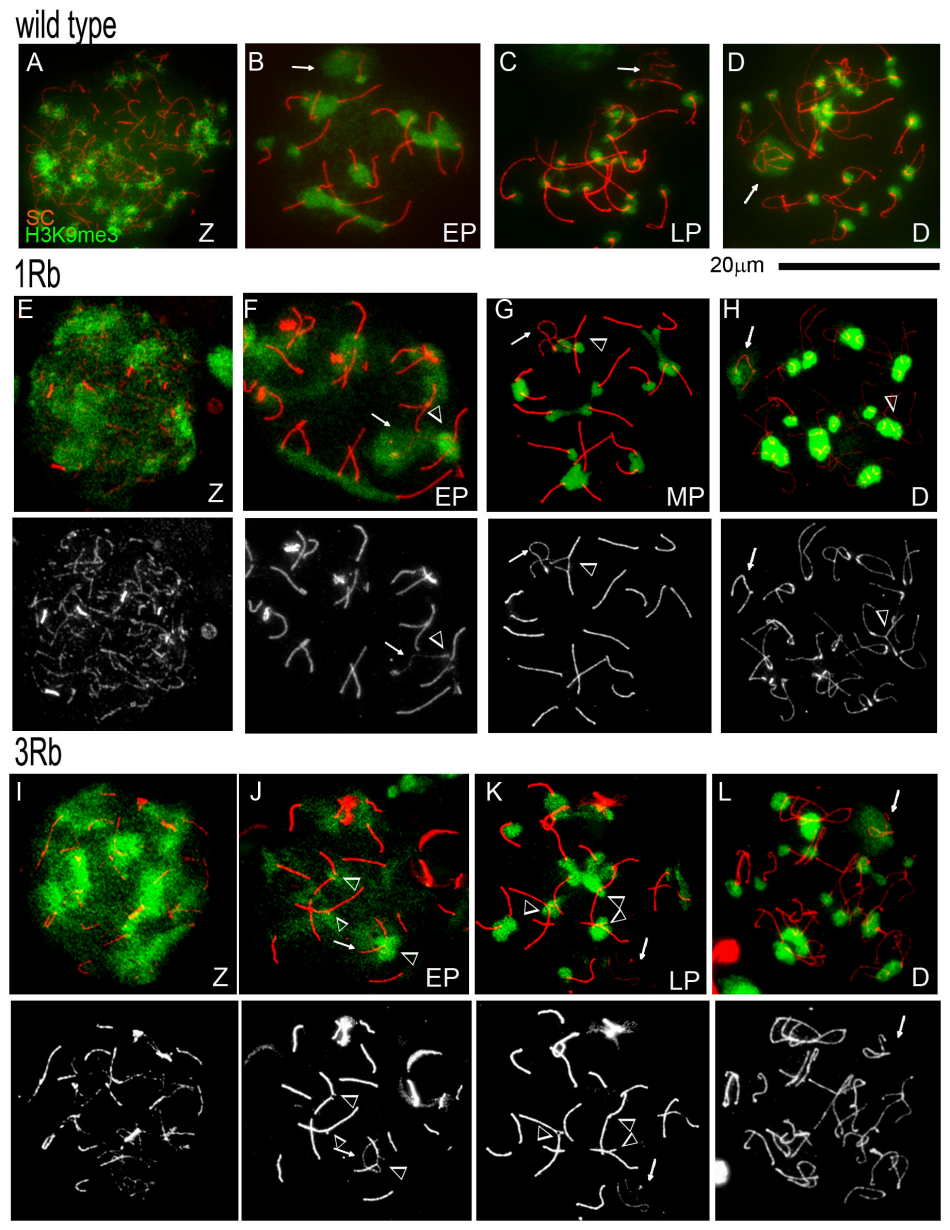
Localization of the heterochromatin mark, histone H3K9me3, at unsynapsed trivalents in meiotic prophase I spermatocytes from carriers of a single or three translocations. Z - zygotene, EP – early pachytene, LP – late pachytene, D – diplotene spermatocytes. Arrows point to the XY bivalents. Arrowheads indicate unsynapsed trivalents. The bottom sets of panels show only SC immunostaining to facilitate the identification of unsynapsed regions and the XY-bivalent. **A-D** - spermatocytes from wild type mice; **E-H** - spermatocytes from carriers of one translocation; **I- L** - spermatocytes from carriers of three translocations. **A**, E and I –zygotene spermatocytes with H3K9me3 enrichment throughout the nucleus. In wild type mice, H3K9me3 is enriched at the sex body in early pachytene (**B**), is lost in mid and late (**C**) pachytene and reappears in diplotene (**D**) spermatocytes. **F**- an unsynapsed autosomal trivalent in early pachytene spermatocytes from a single translocation carrier shows enrichment with H3K9me3 when associated with or in close proximity to the sex body. **G** – an unsynapsed trivalent in a mid pachytene spermatocyte from a single translocation carrier shows distinct H3K9me3 localization at centromeres, but not the rest of the unsynapsed region. The XY-bivalent in the same nucleus shows H3K9me3 enrichment only at the centromere of the X-chromosome. **H** – diplotene spermatocyte from a single translocation carrier with H3K9me3 enrichment at the sex body. **J** – in carriers of three translocations, unsynapsed autosomal trivalents in early pachytene spermatocytes show enrichment with H3K9me3 when associated with or in close proximity to the sex body. **K** –synapsed trivalents in a late pachytene spermatocyte from a carrier of three translocations show H3K9me3 localization at centromeres. The XY-bivalent in the same nucleus shows H3K9me3 enrichment only at the centromere of the X-chromosome. **L** – XY-body enrichment with H3K9me3 in diplotene spermatocytes from carriers of three translocations.

In wild type and single translocation carrier mice, *H3K9me3* was abundant and rather diffuse in zygotene nuclei (Figure 3A, E), localized to the centromeric regions of all chromosomes starting from early pachytene (Figure 3B-D, F-H) and chromocentres of round spermatids; and was seen in elongating spermatids (data not shown) in agreement with other studies [46,42]. H3K9me3 association with the XY bivalent was observed in early pachytene and diplotene nuclei (Figure 3 B, D, F, H). As expected, H3K9me3 was excluded from the XY body in mid and late pachytene spermatocytes (Figure 3 C, G).

The unsynapsed regions of autosomal trivalents showed high enrichment for H3K9me3 in early pachytene spermatocytes (Figure 3F). In mid and late pachytene nuclei, the unsynapsed regions were not enriched for H3K9me3, whereas H3K9me3 foci localized to the centromeres of the chromosomes forming the trivalent (Figure 3G). Similar H3K9me3 localization patterns were observed in carriers of three translocations (Figure 3, I-L). Our data show that H3K9me3 marks the unsynapsed trivalents in early pachytene spermatocytes, and imply that, in the male germ line, H3K9me3 may be a marker of autosomal asynapsis and MSUC in early Pachynema.

**Figure 4:**
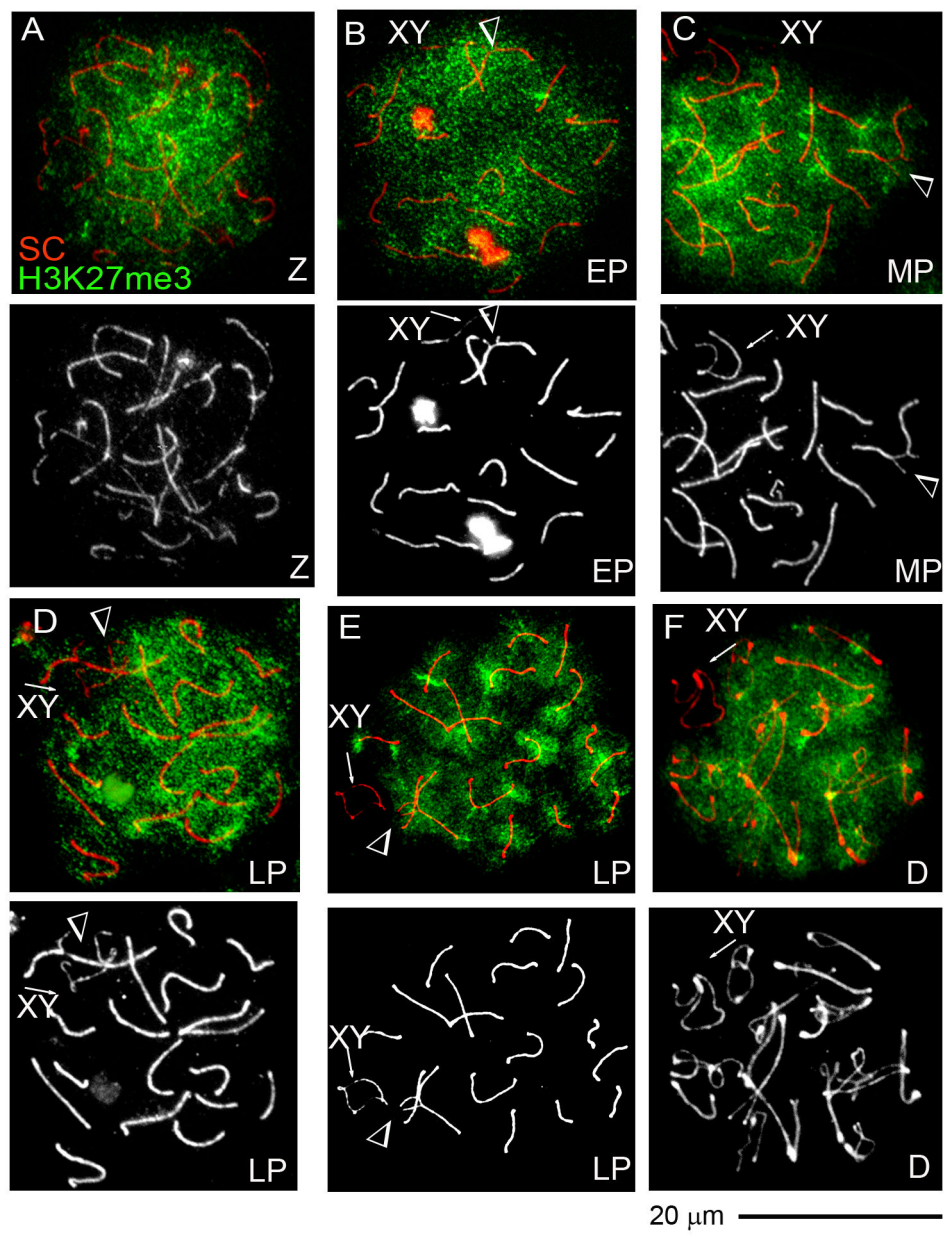
Exclusion of the inactive chromatin mark histone H3K27me3 from unsynapsed chromosomal regions is limited to the XY-bivalent in single translocation carriers. Z - zygotene, EP – early pachytene, LP – late pachytene, D – diplotene spermatocytes. Arrows point to the XY bivalents. Arrowheads indicate unsynapsed trivalents. **A** – Zygotene spermatocyte is enriched for H3K27me3; **B-F** - the H3K27me3 histone mark is excluded from the XY-bivalent in early (**B**) mid (**C**), late (**D**, **E**) pachytene and diplotene (**F**) spermatocytes. However, H3K27me3 is not excluded from the unsynapsed trivalent (**B**, **C** and **E**). **D** - Only one of 15 late pachytene spermatocytes with unsynapsed trivalent showed H3K27me3 exclusion.

*H3K27me3* was detected in all meiotic and post meiotic spermatogenic cells from wild type mice and single translocation carriers (Figure 4 and data not shown). In meiotic prophase I spermatocytes of single translocation carriers, H3K27me3 was excluded from the sex body starting from the mid pachytene stage (Figure 4C-F), as expected. However, H3K27me3 was present at unsynapsed as well as synapsed trivalents in the vast majority of nuclei (Figure 4B, C, E data not shown). In late pachytene spermatocytes, H3K27me3 was excluded from the unsynapsed trivalent in only one out of 15 nuclei (Figure 4D). In this nucleus, the unsynapsed trivalent was tightly associated with the sex body.

Thus, in the vast majority of pachytene spermatocytes, the dynamics of H3K27me3 localization at unsynapsed autosomal regions is similar to that of synapsed chromosomal regions and distinct from that observed at the XY-bivalent.

### Histone H3.3 association with the translocated chromosomes and their homologs in metaphase/anaphase I spermatocytes

**Figure 5:**
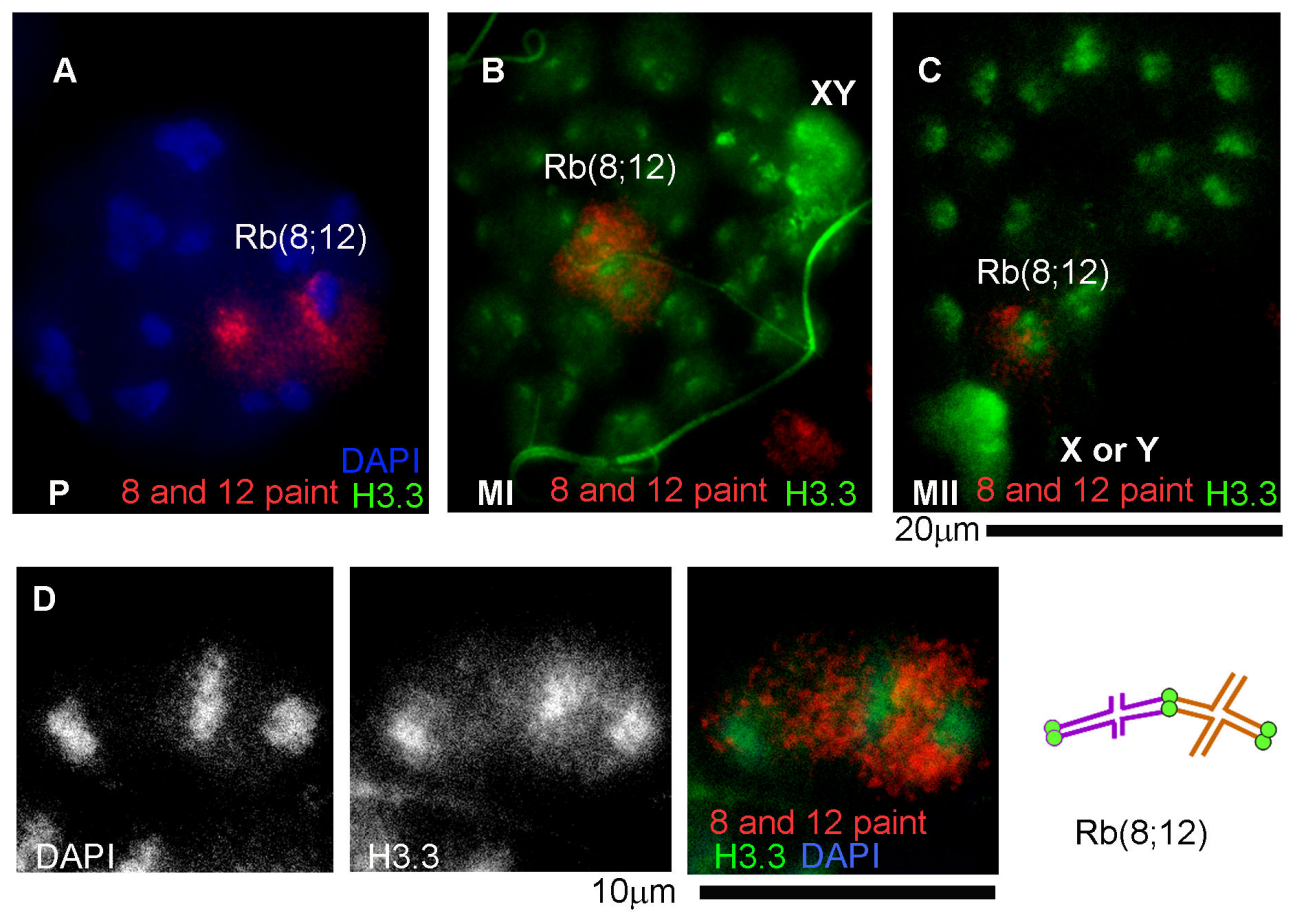
Identification of the Rb(8;12) trivalent in spermatocytes by FISH. **A** –pachytene spermatocyte with a FISH signal corresponding to the Rb(8;12) trivalent, no H3.3 localization is detected; **B** –metaphase/anaphase I spermatocyte with H3.3 enrichment at the sex body; FISH shows the Rb(8;12) trivalent in the center of the nucleus; **C** –metaphase II spermatocyte with H3.3 enrichment at a sex chromosome and only one FISH-positive spot corresponding to Rb(8;12); **D** – structure of the trivalent in a metaphase/anaphase I spermatocyte. The picture has been magnified to show detail. A diagram with the inferred structure is shown on the right.

In the sex body, eviction of nucleosomes that carry the histone H3.1/2 variants and their replacement with histone variant H3.3-containing nucleosomes occurs during the mid-pachytene stage [45]. To evaluate the H3.1/2 nucleosome replacement by H3.3 nucleosomes, immunostaining experiments using an antibody against histone H3.3 phosphorylated at serine 31 (H3.3S31) were conducted. Unlike the antibody used by others [45], this antibody did not detect H3.3S31 in mid or late pachytene spermatocytes but showed intense and specific immunostaining at the sex body in diakinesis, metaphase I, anaphase I and metaphase II spermatocytes (Figure 5 and data not shown). We focused on metaphase/anaphase I spermatocytes and conducted FISH experiments with chromosome painting probes for chromosomes Y (Figure S3), 8 and 12 (Figure 5) to identify the sex body and the trivalent.

**Figure 6:**
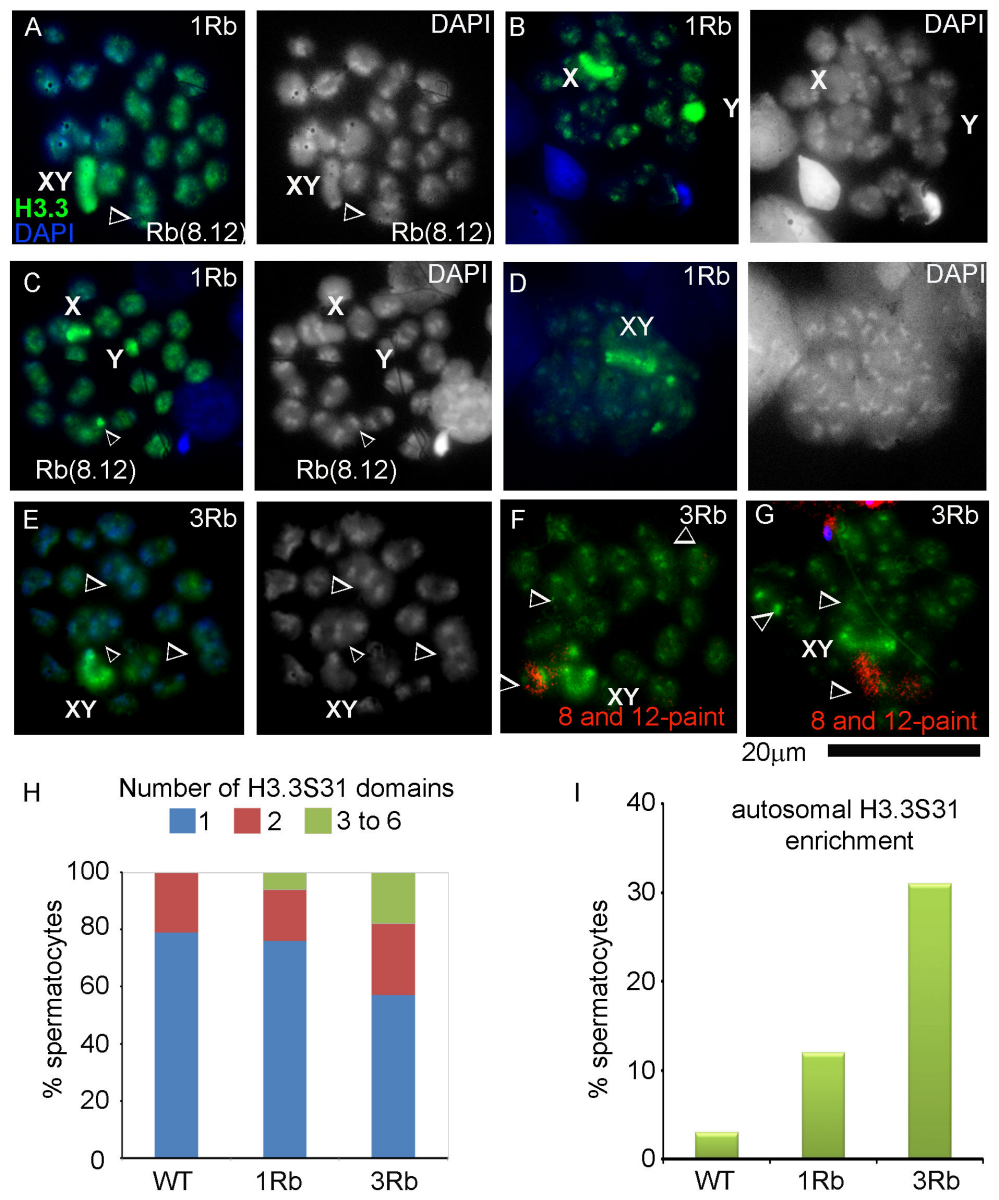
Histone H3.3 marks in metaphase/anaphase I spermatocytes from carriers of one or three Robertsonian translocations. Panels on the left show H3.3S31 immunostaining merged with DAPI staining. Panels on the right show DAPI staining alone. Arrowheads indicate chromosomal trivalents. **A** – a single H3.3S31-enrichment domain in a spermatocyte from a single translocation carrier corresponds to the sex body; **B** - two H3.3S31-enrichment domains correspond to the X and Y univalents; **C, D** - three H3.3S31-enrichment domains correspond to XY (D) or X and Y separately (C) and autosomal centromeric regions. **E** - a single H3.3S31-enrichment domain in a spermatocyte from a carrier of three translocations corresponds to the sex body. **F-G** - FISH for chromosomes 8 and 12 (red) shows presence (**F**) or absence (**G**) of co-localization of the Rb(8;12) trivalent with the autosomal H3.3S31 enriched domain in carriers of three translocations. **H** – distribution of spermatocytes with one, two or more than two H3.3S31 enrichment domains in wild type congenic mice without translocations, heterozygous Rb(8;12) carriers and heterozygous (Rb(1;3), Rb(8;12) and Rb(9;14) carriers. **I** – percent spermatocytes with autosomal H3.3S31 enrichment in heterozygous carriers of translocations compared to wild type congenic males.

If the trivalents were enriched with H3.3, they would appear as intense signals distinct from the sex chromosomes. Therefore, we counted the number of metaphase/anaphase I nuclei with one, two or more H3.3-positive domains. *In single translocation carriers*, 76% of metaphase/anaphase I nuclei (106 nuclei counted) had one H3.3S31-enriched domain, the sex body (Figure 6A); and 24% contained two or more H3.3S31-enriched domains (Figure 6B-D). In nuclei with more than one H3.3S31 enriched domains, often, two of them corresponded to the X and Y univalents (Figure 6C and D). In 13 of 106 (12%) counted nuclei, the H3.3-enriched domains localized to autosomes (Figure 6C, D and I). Thus, only 12% or a smaller proportion of metaphase/anaphase I spermatocytes carry the H3.3 histone mark associated with MSUC on autosomes.

*In wild type mice* (congenic B6.SPRET7MOLF12 mice without translocations), 79% of metaphase/anaphase I nuclei (99 counted) had one H3.3S31-enriched domain and 21% contained two H3.3S31-enriched domains. No nuclei with three or four H3.3S31-enriched autosomal domains were observed. Only three nuclei (3%) contained H3.3S31-enriched domains that localized to autosomes (Figure 6I). Therefore, a significantly higher proportion of nuclei in translocation carriers have H3.3S31 enrichment at autosomes (presumably at trivalents in Robertsonian translocation carrier mice) compared to wild type mice (Fisher’s exact test, p=0.01).

We conclude that replacement of histone H3.1/2 nucleosomes by nucleosomes that carry the histone variant H3.3 occurred in 12% or a smaller proportion (e.g. 9%, if 3% of these H3.3S31 positive signals represent random events as in the wild type mice) of spermatocytes, which is consistent with the small proportion of spermatocytes with histone γH2AX localization at unsynapsed trivalents at the late pachytene stage. Conversely, this small proportion is also in agreement with persistence of H3K27me3 at unsynapsed autosomal trivalents in the vast majority of pachytene spermatocytes.

To determine if the number of translocations influenced the proportion of nuclei with H3.3S31 enriched autosomes, we analyzed the H3.3 enrichment in spermatocytes from carriers of the three translocations. *In carriers of three translocations*, 57% of metaphase/anaphase I nuclei (175 nuclei counted) had one H3.3S31-enriched domain, the sex body, (Figure 6E and H); and 43% contained two or more H3.3S31-enriched domains (Figure 6F, G and H). In 54 of the 175 nuclei, (31% of all counted nuclei), the H3.3S31-enriched domains localized to autosomes (Figure 6F, G and I), a higher proportion than in carriers of a single Robertsonian translocation (Fisher’s exact test, p=0.0005). The number of H3.3S31 enriched autosomal domains varied among nuclei (range 1 to 6) with one and two domains per nucleus being the most common occurrence (found in 26 and 21 nuclei, respectively). Among those with two H3.3S31 enrichment domains, the most common pattern was the presence of two domains in the same trivalent (Figure 6F and G). Hence, an increased proportion of spermatocytes show autosomal H3.3S31 enrichment in carriers of three translocations. However, we did not find nuclei with H3.3S31 enrichment at all three trivalents. This pattern suggests that H3.3 enrichment at different trivalents occurs independently in each spermatocyte.

If H3.3 is an epigenetic mark of MSUC, then our data suggest that MSUC persists in 12% of spermatocytes of the single translocation carriers and 31% of spermatocytes from three translocation carriers, i.e. with a larger number of translocations a larger number of spermatocytes are affected by meiotic silencing.

## Discussion

### Frequency of MSUC varies between prophase and metaphase I spermatocytes of Robertsonian translocation carriers

Our study shows an efficient early response to asynapsis and loading of γH2AX and BRCA1 at unsynapsed autosomal regions of the trivalents in Robertsonian translocation carriers. In contrast to sex chromosomes, however, non-homologous centromeres of autosomal trivalents succeed in synapsis in the vast majority of late pachytene spermatocytes in agreement with previous reports [10,23,25,27,28]. This parallels the loss of γH2AX and BRCA1 from the trivalents in the vast majority of spermatocytes by the late pachytene stage.

Similarly to γH2AX and BRCA1, the mark of constitutive heterochromatin, histone H3K9me3, is often enriched at unsynapsed trivalents in early pachytene spermatocytes. The mark of facultative heterochromatin, H3K27me3 that is normally excluded from the sex body starting from the mid pachytene stage [45], is however present at the unsynapsed trivalents throughout pachytene. Interestingly, H3K27me3 is not excluded from the unsynapsed single X chromosome in the oocytes of XY sex-reversed females, where sex body formation does not occur [47]. These lines of evidence point to a specific function of the sex-body formation in the exclusion of H3K27me3.

At later stages, histone variant H3.3 that replaces histones H3.1 and H3.2 on the unsynapsed sex chromosomes, is associated with autosomes in the minority (12%) of metaphase/anaphase I spermatocytes in single translocation carriers. Since the exclusion of H3K27me3 from the sex body is due to a replacement of histone variants H3.1 and H3.2 by histone H3.3 [45], lack of H3.3 accumulation is consistent with the persistence of H3K27me3 at unsynapsed trivalents in most spermatocyte nuclei from single translocation carriers.

In spermatocytes of carriers of the three translocations Rb(1;3), Rb(8;12), and Rb(9;14), the proportion of nuclei with H3.3 localization to the trivalents showed a nearly three-fold increase. Most nuclei had only one H3.3 positive trivalent suggesting that MSUC occurred independently at different trivalents. These data indicate that the proportion of spermatocytes with meiotic silencing depends on the number of translocations.

In single translocation carriers, we observed autosomal H3.3S31 enrichment in 12% of metaphase /anaphase I spermatocytes. This proportion is not statistically different from the proportion of late pachytene spermatocytes that carry unsynapsed trivalents with or without histone γH2AX localization (p= 0.2117, Fisher’s exact test). Furthermore, in our recent study of the same single Rb(8;12) translocation, we detected a DNA methylation defect at the *H19* imprinting control region in about 10% of mature spermatozoa [26]. We hypothesized that the defect resulted from meiotic silencing of the *Dnmt3a* gene that is located at position 3Mb on chromosome 12, i.e. near the centromere. The 10% estimate of spermatocytes with a silent *Dnmt3a* gene is consistent with the 12% of metaphase/anaphase I spermatocytes harboring H3.3-enriched autosomes in single translocation carriers.

Thus, if both γH2AX and H3.3S31 are markers of MSUC, then meiotic silencing of the unsynapsed portions of a given trivalent initially occurs in the vast majority of early pachytene spermatocytes, but with meiotic progression and non-homologous synapsis, epigenetic marks of MSUC remain in only a small proportion of spermatocytes by the time they reach the late pachytene stage and then metaphase I. This is consistent with two phases of meiotic silencing, a reversible and an irreversible one, demonstrated for the mouse sex chromosomes [48]. We hypothesize that meiotic silencing and heterochromatinization of unsynapsed trivalents found in the majority of early pachytene nuclei represent the reversible phase, while the irreversible phase of meiotic silencing of unsynapsed trivalents occurs in a relatively small fraction of spermatocytes.

An alternative explanation of the decline in the number of spermatocytes with γH2AX and BRCA1-positive trivalents, is the elimination of spermatocytes with autosomal MSUC during the mid pachytene stage of meiosis. Under such a scenario, only spermatocytes with synapsed trivalents or γH2AX and BRCA1-negative unsynapsed trivalents would reach the late pachytene stage. This alternative explanation however is not supported by data from heterozygous carriers of eight Robertsonian translocations, where no major loss of spermatocytes is observed before the metaphase stage of meiosis [25], nor does it explain the fact that different translocations have different impact on spermatogenesis. Therefore, at least for the translocations that were used in our study, we favor the scenario where the majority of spermatocytes with unsynapsed trivalents are not eliminated during the pachytene stage.

Based on the hypothesis that phenotypic outcomes of Robertsonian translocations may differ depending on the function of genes residing near the translocation breakpoint, e.g., the centromeric regions in Robertsonian translocations [15], we propose that the timing of MSUC may also influence the phenotype. If the gene is critical for meiotic progression at the early pachytene stage when most trivalents are not synapsed, with its meiotic silencing occurring in nearly all spermatocytes, the translocation is likely to cause a significant phenotypic effect, such as spermatogenic failure and infertility of the carrier. However, if the gene product is essential after metaphase I, when MSUC occurs in a small proportion of spermatocytes, the translocation will hardly have a discernable phenotypic outcome in the carrier, but may cause anomalies in a small proportion of his offspring due to aneuploidies or abnormal epigenetic reprogramming. Indeed, it has been hypothesized that in mammalian germ cells histone variant H3.3 may escape the genome-wide replacement of histones by protamines and act as a transgenerational memory mark transmitting epigenetic information from the father to his offspring [49,50]. Moreover, experimental evidence points to a critical role that histone variant H3.3 plays in the formation of heterochromatin on paternal chromosomes of mouse zygotes [51]. It is therefore a reasonable conjecture that meiotic H3.3 enrichment at unsynapsed regions, if transmitted to embryos, may cause abnormal heterochromatinization, affect the functioning of the paternal genome and thereby compromise embryonic development. To establish whether such a transgenerational epigenetic inheritance affects the progeny of translocation carriers, detailed analysis of chromatin of the germ cells and embryos from translocation carriers is necessary.

### Relative dynamics of γH2AX and BRCA1 recruitment and retention at unsynapsed trivalents

BRCA1 immunolocalization shows two types of BRCA1-positive unsynapsed trivalents: those with discrete BRCA1-positive foci and those with high BRCA1 enrichment. Discrete BRCA1 foci are observed predominantly in early pachytene and look similar to the discrete foci found in zygotene spermatocytes (Figure 2A). High BRCA1 enrichment is seen in both early and mid pachytene spermatocytes, whereas the BRCA1 signal was not detected in late pachytene spermatocytes, suggesting that BRCA1 was removed from the unsynapsed axes in the majority of late pachytene spermatocytes. We therefore hypothesize that the discrete foci correspond to earlier steps of BRCA1 recruitment to unsynapsed trivalents and that BRCA1 is recruited/accumulated at unsynapsed trivalents when γH2AX is already in place.

In somatic cells, phosphorylation of histone γH2AX at and near the DSBs is one of the first epigenetic events following DSB formation (reviewed in [52]) and is prerequisite for the recruitment and retention of the BRCA1-A complex that is essential for DSB repair [53] (reviewed in 52,54,55). In the mammalian meiotic prophase, however, the proposed sequence of events is more complex and different from that established for somatic cells [8,19,48,56]. Briefly, ATR regulates localization of BRCA1, BRCA1 is recruited to unsynapsed sex chromosomal axes, where recruits certain members of the BRCA1-A complex [56] and directs localization of ATR; while ATR, in turn, phosphorylates H2AFX [8,19,48]. We observed BRCA1 foci at the unsynapsed trivalents at the early pachytene (Figure 2, B and I) and increased BRCA1-enrichment as spermatocytes progress towards the mid pachytene stage (Figure 2, C and F). Therefore, our data for the unsynapsed trivalents are not fully consistent with the time line of BRCA1 localization at the sex chromosomes but compatible in the timing aspect with the sequence of events in somatic cells. We propose that in the unsynapsed regions of the trivalents, BRCA1 is present at single foci at the onset of Pachynema. Persistence of asynapsis in early pachytene spermatocytes initiates a new wave of recruitment of BRCA1 to unsynapsed regions; and this may lead to or coincide with non-homologous synapsis and DSB repair. Once the DSBs are repaired, BRCA1 and γH2AX are lost from the synapsed regions in mid and late pachytene spermatocytes.

## Conclusions

Collectively our data suggest that the dynamics of response to asynapsis in Robertsonian translocations differs from the response to sex chromosomal asynapsis in the male germ line. The recently reported asynchrony between the sex chromosomes and unsynapsed autosomes with respect to the timing of transcriptional silencing [18] supports this conclusion. These differences may stem from several sources including non-homologous synapsis of trivalents or formation of a special compartment, the sex body, around the XY bivalent, but not the unsynapsed trivalents. Formation of the sex body in turn may contribute to the exclusion of H3K27me3 from the unsynapsed sex chromosomes. Further studies are necessary to clarify the full extent of the differences in the response to asynapsis between sex chromosomes and autosomal translocations, its dependence, if any, on the type of chromosomal rearrangement and whether these differences apply to Robertsonian translocations only or may be extrapolated to autosomal asynapsis in general.

## Supporting Information

Figure S1 **Configurations of chromosomal axes in pachytene spermatocytes from carriers of Robertsonian translocations.** **A** - Unsynapsed and synapsed trivalents. **B** – Entangled unsynapsed trivalents and sex chromosomes are often observed in carriers of the three translocations. Arrows point to the XY bivalents. Arrowheads indicate unsynapsed trivalents. The associations between chromosomes make the staging difficult. The left panel shows combination of γH2AX, SYCP3 and DAPI staining in two pachytene nuclei with the sex chromosomes associated with unsynapsed trivalents. The right panel shows the SYCP3 staining alone for axes visualization. The top nucleus contains a large area of γH2AX enrichment; three unsynapsed trivalents, two of which interact with, presumably, the XY bivalent. An unsynapsed univalent is also visible. However, unambiguous identification of the XY bivalent and the stage of pachytene are not possible for this nucleus. The bottom nucleus also shows association between the Y chromosome and the unsynapsed trivalent. Based on the configuration of the XY bivalent, it is a mid pachytene stage spermatocyte. However, two of the three trivalents are not readily identifiable.
(TIF)

Figure S2 **Exclusion of POLII from centromeric regions of autosomes.** **A**- a late pachytene spermatocyte from a single translocation carrier; **B**- mid and **C**- late pachytene spermatocytes of carriers with three translocations. Arrows point to the XY bivalents. Arrowheads indicate unsynapsed trivalents.
(TIF)

Figure S3 **Localization of the sex body and H3.3S31 enrichment in metaphase/anaphase I spermatocytes.** Y-chromosome-specific FISH was conducted after the immunostaining with anti-H3.3S31 antibodies. **A** - nucleus with co-localization of the Y-paint (red) and H3.3S31 enrichment (green) in the sex body. **B** - nucleus with X and Y chromosomes as separate domains.
(TIF)
