# Supplementary figures and images for: Dynamics of Response to Asynapsis and Meiotic Silencing in Spermatocytes from Robertsonian Translocation Carriers
Source: PLoS One. 2013 Sep 16;8(9):e75970. doi: 10.1371/journal.pone.0075970 (PMC3774740; doi:10.1371/journal.pone.0075970)

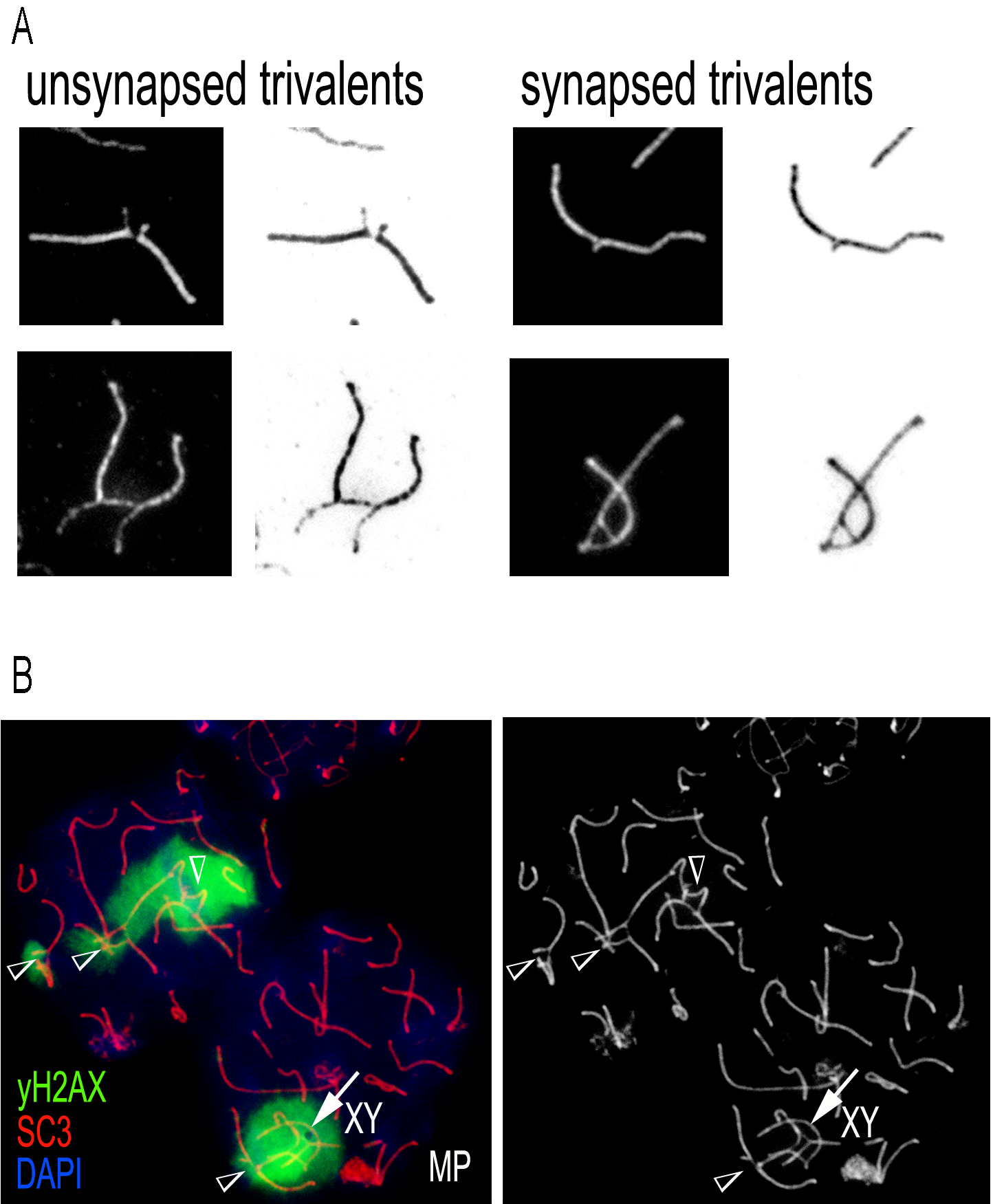

Supplement: Figure S1 — Configurations of chromosomal axes in pachytene spermatocytes from carriers of Robertsonian translocations. A - Unsynapsed and synapsed trivalents. B – Entangled unsynapsed trivalents and sex chromosomes are often observed in carriers of the three translocations. Arrows point to the XY bivalents. Arrowheads indicate unsynapsed trivalents. The associations between chromosomes make the staging difficult. The left panel shows combination of γH2AX, SYCP3 and DAPI staining in two pachytene nuclei with the sex chromosomes associated with unsynapsed trivalents. The right panel shows the SYCP3 staining alone for axes visualization. The top nucleus contains a large area of γH2AX enrichment; three unsynapsed trivalents, two of which interact with, presumably, the XY bivalent. An unsynapsed univalent is also visible. However, unambiguous identification of the XY bivalent and the stage of pachytene are not possible for this nucleus. The bottom nucleus also shows association between the Y chromosome and the unsynapsed trivalent. Based on the configuration of the XY bivalent, it is a mid pachytene stage spermatocyte. However, two of the three trivalents are not readily identifiable. (TIF) [file pone.0075970.s001.tif]

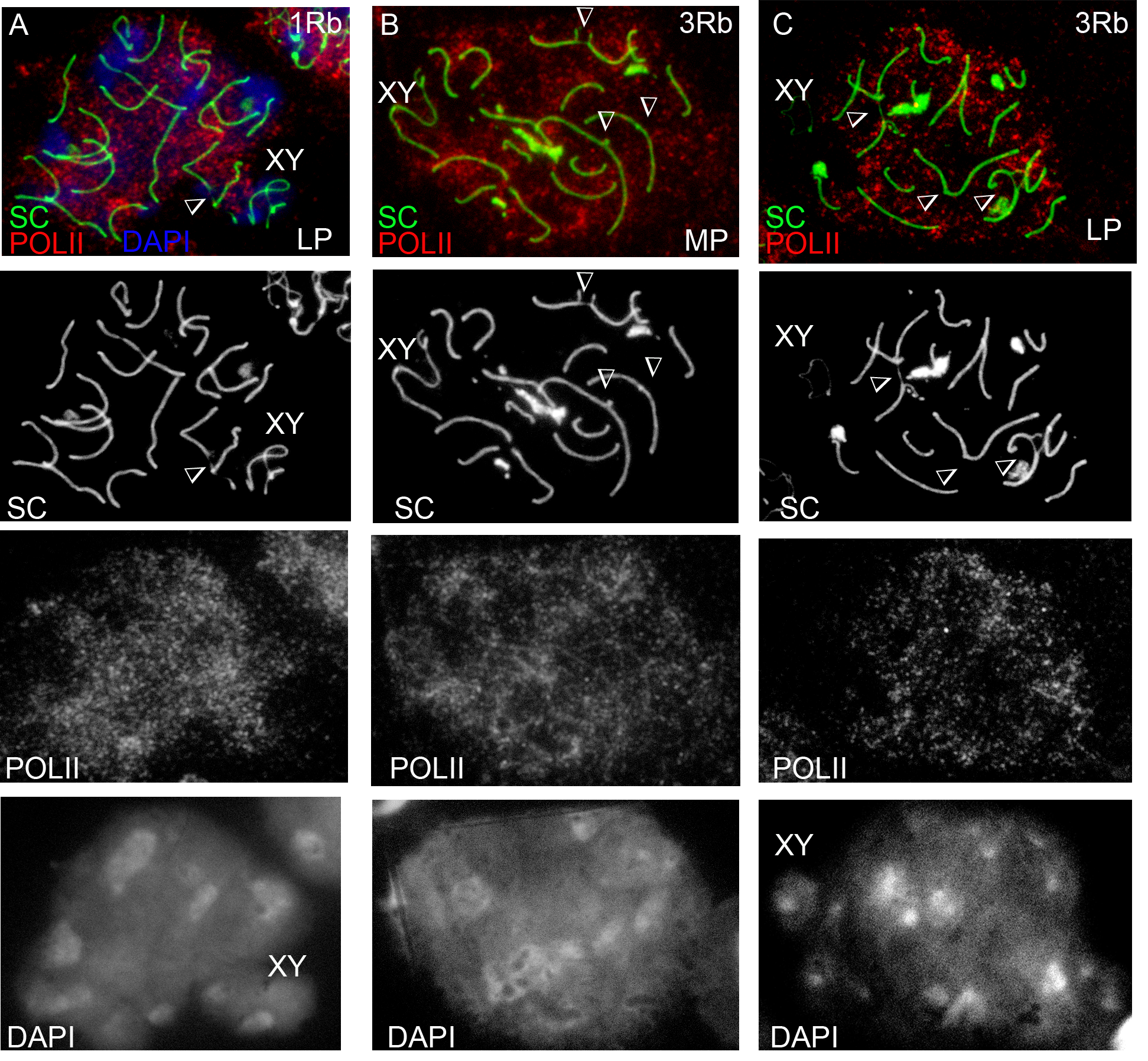

Supplement: Figure S2 — Exclusion of POLII from centromeric regions of autosomes. A- a late pachytene spermatocyte from a single translocation carrier; B- mid and C- late pachytene spermatocytes of carriers with three translocations. Arrows point to the XY bivalents. Arrowheads indicate unsynapsed trivalents. (TIF) [file pone.0075970.s002.tif]

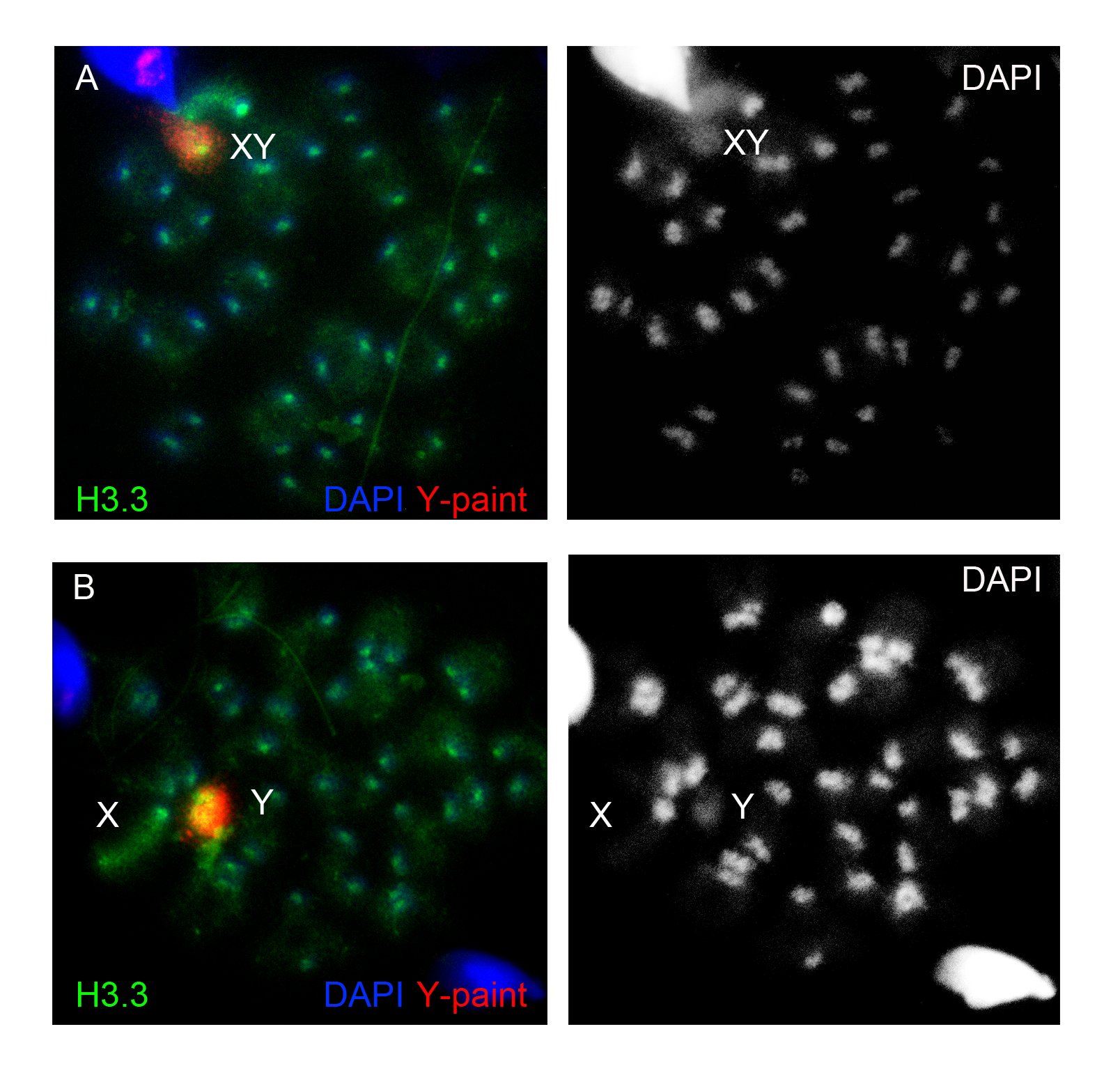

Supplement: Figure S3 — Localization of the sex body and H3.3S31 enrichment in metaphase/anaphase I spermatocytes. Y-chromosome-specific FISH was conducted after the immunostaining with anti-H3.3S31 antibodies. A - nucleus with co-localization of the Y-paint (red) and H3.3S31 enrichment (green) in the sex body. B - nucleus with X and Y chromosomes as separate domains. (TIF) [file pone.0075970.s003.tif]
